# Smart Probes for Ultrasensitive and Highly Selective Sensing of Homocysteine over Cysteine Based on Multi-Cooperative Effects by Using Gold Nanoparticles

**DOI:** 10.3390/molecules30061309

**Published:** 2025-03-14

**Authors:** Manman Sun, Peihao Zhang, Zeze Xie, Pengcheng Zhang, Zhendong Li, Zhiguang Yang, Hongyu Chen

**Affiliations:** 1 College of Physics and Telecommunication Engineering, Zhoukou Normal University, Zhoukou 466001, China; 2 Henan Key Laboratory of Rare Earth Functional Materials, College of Chemistry and Chemical Engineering, Zhoukou Normal University, Zhoukou 466001, China

**Keywords:** colorimetric sensing, multi-cooperative effects, homocysteine, high sensitivity, high selectivity, gold nanoparticles

## Abstract

Homocysteine (Hcy) is a biothiol that plays a vital role in many physiological processes and is involved in a variety of diseases. However, it is significantly difficult to discriminate Hcy from cysteine (Cys) due to their similar chemical structures (only one methylene difference) and reactivity. In this study, a novel nanosensor was proposed to discriminate Hcy from Cys with multi-cooperative effects by using gold nanoparticles (AuNPs). The discrimination effect for Hcy originates from the interaction difference of the hydrogen bonding, steric hindrance, and carbon chain length in Hcy and Cys with AuNPs. Under the best conditions, this nanosensor has two unique advantages. Firstly, the sensor exhibits high sensitivity with detection limits of 0.1 μM through naked-eye determination and 0.008 μM through UV−vis spectroscopy analysis. Secondly, the sensor showed superior selectivity for Hcy over the other 16 natural amino acids (biothiol-containing Cys and glutathione (GSH)), and it is the first time to clearly distinguish Hcy from Cys (the Cys concentration is 40 times higher than Hcy). Furthermore, the system was further employed to detect Hcy in human serum, and the result was in agreement with that tested by clinicians via enzymatic assays, with acceptable recovery.

## 1. Introduction

As a crucial amino acid containing a free thiol moiety, homocysteine (Hcy) has many important roles within physiological matrices [1]. Normal concentrations of Hcy in blood plasma range from 5 to 15 µM [2]. An elevated level of Hcy in the blood is a strong indicator for cardiovascular diseases [3], stroke, and arteries or venous thrombosis [4]. In addition, Hcy has also been reported to be associated with neurodegenerative disorders, such as neural tube defects, Alzheimer’s disease, and other cognitive impairments [5,6]. Thus, the quantitative and qualitative detection of Hcy become more and more important for disease prediction and early diagnosis. Current methods for determining Hcy levels mainly include immunoassay [7], high-performance liquid chromatography with MS [8], capillary electrophoresis [9] and enzymatic assays [10]. Even though these techniques offer high accuracy, they typically requires staff with a certain skill level, tedious sample pretreatment, sophisticated instrumentation, time, and high operating costs for the analysis, which makes it unsuitable for on-site trials and household testing. Optical probes, especially small molecular fluorescent probes, which possess advantages of high sensitivity, low cost, convenience, and non-invasiveness, are therefore very attractive for biothiol (Hcy, cysteine (Cys), and glutathione (GSH)) sensing. As a result, much effort has been devoted to the design and fabrication of fluorescent sensors for thiol recognition [11,12,13,14,15,16]. The processes of detecting thiols generally involve some specific reactions between probes and thiols, such as cyclization with aldehyde, Michael addition, cleavage reaction by thiols, metal complexes’ oxidation–reduction, metal complexes’ displacement coordination, and others. Although many fluorescent probes for thiols have been developed, there are fewer reports of molecular sensors that are selective for Hcy compared to those enabling Cys or GSH selectivity [17]. Hcy is a homologue of Cys with only one additional methylene (–CH_2_–) group, which makes it challenging to differentiate these two. Recently, very few studies have been reported to solve this problem. For instance, Strongin et al. developed a system for discrimination detection of Hcy and Cys based on the difference in cyclization rate between the two analytes and the probe [18]. Zhao et al. reported an NIR fluorescent probe containing a partially exposed aldehyde group for the detection of Hcy over Cys [19]. Even though these probes respond better to Hcy than Cys, they are still unable to detect Hcy levels in human serum, because the level of Cys in human serum is typically 15–20 times higher than that of Hcy, which impacts the detection of Hcy in serum. Our group proposed a novel fluorescent probe with four potential reaction sites for simultaneous and rapid sensing of Cys, Hcy, and GSH from three emission channels [20]. Although the multiple binding site probe can effectively distinguish Cys, Hcy, and GSH, it was still not appropriate for the detection of Hcy in serum because the difference between the emissions with Hcy and GSH was small (30 nm). On the other hand, both single site probes and multiple binding site probes require complicated preparation procedures, relatively long response times, and a portion of organic solvents, which limits their practical application, especially in biological systems.

Nanoparticle probes have been successful in detection selectivity of biothiols over other amino acids owing to their excellent photochemical stability, good water solubility, chemical inertness, low toxicity, and favorable biocompatibility [21,22,23]. Lin et al. used europium-decorated graphene quantum dots coordinated with Cu^2+^, where the Cu^2+^ was the quencher, and the quantum dots’ fluorescence was reactivated by L-cysteine [21]. This way is convenient and relatively quick, but it lacks specificity. Tseng et al. developed a selective-detection system for Hcy based on the combination of fluorosurfactant-capped gold nanoparticles (FSN-AuNPs) and o-Phthaldialdehyde [22]. This method manifested high selectivity. However, it involved long reaction times (1.6 h) and relatively complex separation steps. Wang et al. designed a novel aldehyde-functionalized metal–organic framework sensor for discriminating Hcy from natural amino acids and even thiol-related peptides (GSH) [23]. Liu et al. constructed a colorimetric sensor array based on a porphyrin-modified CoMoO₄ nanozyme for the selective detection of Hcy [24]. Both of the above-mentioned probes can selectively detect Hcy in the presence of Cys at the same concentration. However, the concentration of Cys in serum is usually much higher than that of Hcy, which limits the practical application of these probes. Thus, selective analysis of Hcy in human serum with a nanoprobe would be highly valuable but even more challenging.

**Scheme 1:**
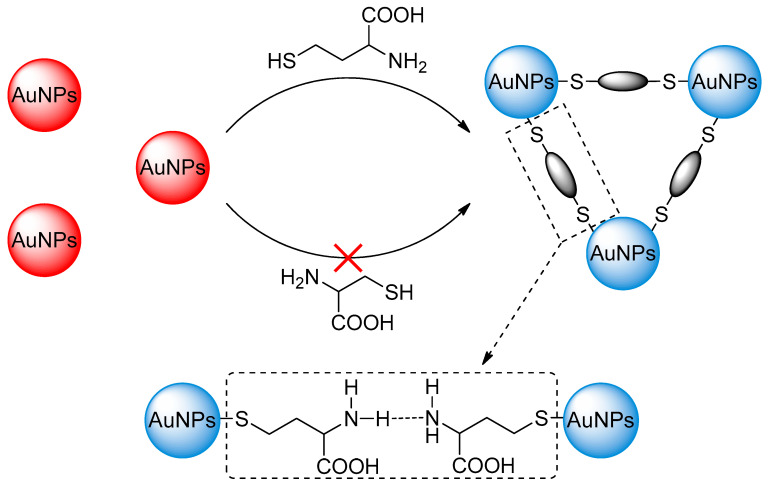
Schematic illustration of the high selectivity detection of Hcy based on multi-cooperative effects by using AuNPs.

In this work, we have found that the aggregation of AuNPs can be selectively triggered by Hcy over natural amino acids and thiol-related peptides (Cys and GSH) in PBS buffer at pH 5.0. As shown in Scheme 1, AuNPs were aggregated because of hydrogen bonding between the amino groups on a pair of particles. It is interesting to note that under our experimental conditions, one methylene (–CH_2_–) difference between Cys and Hcy can cause a very different effect in the aggregation of AuNPs due to the interaction difference of the hydrogen bonding, steric hindrance, and carbon chain length. The sensing mechanism is also proved through our experiments and discussions. The designed method shows many advantages, including briefness, rapidness, high sensitivity, and excellent selectivity, and it can directly recognize the concentration of Hcy through visual observation. In addition, this study not only exemplifies the use of target analytes’ physical properties on nanoparticle surfaces to enhance nanosensor performance but also provides new design ideas for constructing other nanosensors. Furthermore, it offers a novel strategy for the specific detection of Hcy, which reveals great potential for the biomarker-specific detection of early disease diagnosis.

## 2. Experimental Details

### 2.1. Materials and Apparatus

Hcy, GSH, Cys, glycine, serine, asparagine, glutamic acid, lysine, tryptophane, cystine, threonine, histidine, alanine, proline, and chloroauric acid (HAuCl_4_) were purchased from Sigma-Aldrich (Shanghai, China). Sodium borohydride (NaBH_4_), sodium citrate, amifostine thiol dihydro chloride, mercapto acetic acid (TGA), cyseamine, penicillamine (Pen), 4-aminothiopheno (amin), and 3-mercaptopropionic acid (MPA) were obtained from Aladdin (Shanghai, China). All of the chemical reagents and solvents used in our experiment were of analytical grade and used directly without further purification. The aqueous solution was prepared using distilled water purified using a Millipore-Q system with a resistance of 18.2 MΩ cm. Clinical human serum samples were provided by Changsha Fourth Hospital (Changsha, China). Transmission electron microscopy (TEM) images were carried out using a JEOL-1230 TEM (JEOL, Tokyo, Japan) to characterize the shape and size of AuNPs. UV–vis absorption spectra of AuNPs were collected on a UV-2450 spectrophotometer (Shimadzu Co., Kyoto, Japan). Zeta potentials of AuNPs were measured using Nano-ZS Zetzsozer ZEN3600 (Malvern Instruments Ltd., Malvern, UK).

### 2.2. Synthesis of AuNPs

All glassware used in the preparation of AuNPs was immersed in freshly prepared aqua regia for several hours and then rinsed with ultrapure water before use. AuNPs were synthesized through a modified method for reducing HAuCl_4_ using citric acid [25,26]. Typically, we added 300 mL of ultrapure water to a 500 mL Erlenmeyer flask and brought it to a boil. Then, 1.06 mL HAuCl_4_ (5 mM) was added to the boiling ultrapure water and vigorously stirred. After 1 min, we added 3.6 mL of trisodium citrate solution (0.0366 M). The mixed solution was boiled for 10 min, followed by stopping heating and stirring for another 15 min. The color of the solution changed from light yellow to burgundy, and it was stored in the refrigerator (4 °C) for further use after the solution was cooled. The concentration of the AuNPs was estimated according to Beer’s law.

### 2.3. Detection of Hcy

Hcy detection was realized as follows: 200 μL of AuNPs (1 nM) was added into a phosphate buffer (5 mM, pH 5.0) containing different concentrations of Hcy in a 2 mL tube, and the mixture was incubated at room temperature (25 ± 1.0 °C) for 20 min. The final volume was 500 μL. The UV–vis absorption spectra of the resulting solution were measured. The selectivity of the sensing system to Hcy was investigated by using other biomolecules or common ions instead of Hcy.

### 2.4. Real Sample Preparation and Analysis

Human serum samples were obtained from the Changsha Fourth Hospital (Changsha, China). Serum samples were pretreated to eliminate all protein interference and improve the recovery. Then, 3 mL of trichloroacetic acid (15 wt%) was introduced into 1 mL of serum to destroy the activity of the protein in the serum and precipitate it from the solution. The mixture was then centrifuged at 13,000 rpm for 15 min to remove the precipitate. The supernatant was obtained and adjusted to pH 7.0 using NaOH solution. Afterwards, it was diluted 4 times using phosphate buffer (PBS, 5 mM, pH 5.0) before measurement. Finally, the Hcy concentration in these samples was analyzed through our method, and then a certain concentration of Hcy was added to these samples to check the percentage recovery.

## 3. Results and Discussion

### 3.1. Choice of Materials

The size of AuNPs affected the analytical performance of the sensing system. To assess the impact of AuNPs’ size on sensing performance, AuNPs of different sizes were firstly synthesized. Then, under the same experimental conditions, the specificity of different-sized AuNPs in recognizing Hcy was tested. As shown in Figure S1A, when the AuNPs were small (average diameter of 11.5 nm), there was no significant interaction between AuNPs and Hcy (Figure S2A). When the AuNPs (Figure S1B) were large (average diameter of 31.6 nm), they failed to specifically recognize Hcy (Figure S2B), as AuNPs of this size interacted well with both Hcy and Cys. Only when the AuNPs’ size was around 22.7 nm (Figure S3) did the developed sensing system exhibit high sensitivity and selectivity for Hcy recognition (Figure S4). All of the above results indicate that selecting 22.7 nm AuNPs for constructing the sensing system is appropriate.

### 3.2. Characteristics of AuNPs

AuNPs were characterized through TEM and zeta potential. It can be seen from Figure S3A that the AuNPs are spherical in shape and uniform in size. The average size is 22.7 nm in diameter by counting about 100 particles (Figure S3B). Figure S5 shows that the zeta potential of dispersed AuNPs was −13.2 mV, which is mainly because AuNPs were stabilized by citrate ions, and a negative electrostatic layer was formed on AuNPs such that the nanoparticles are uniformly and stably dispersed in aqueous solution.

### 3.3. The Working Principle of the Proposed Method

Under our experimental conditions, the aggregation force of AuNPs induced by Hcy is much greater than that by Cys. Based on this phenomenon, the interference of Cys can be eliminated so that the selective detection of Hcy can be realized. There is no literature regarding the role of amino and carboxyl groups in the aggregation of AuNPs in Hcy and Cys molecules or any elucidation of the phenomenon through which Hcy and Cys molecules show very different forces and cause the aggregation of AuNPs with such similar structures. Starting with the nuances of the structures of the two molecules, this paper researches the reasons for the difference forces between the two and the AuNPs from the aspects of the group effect and carbon chain length.

**Figure 1:**
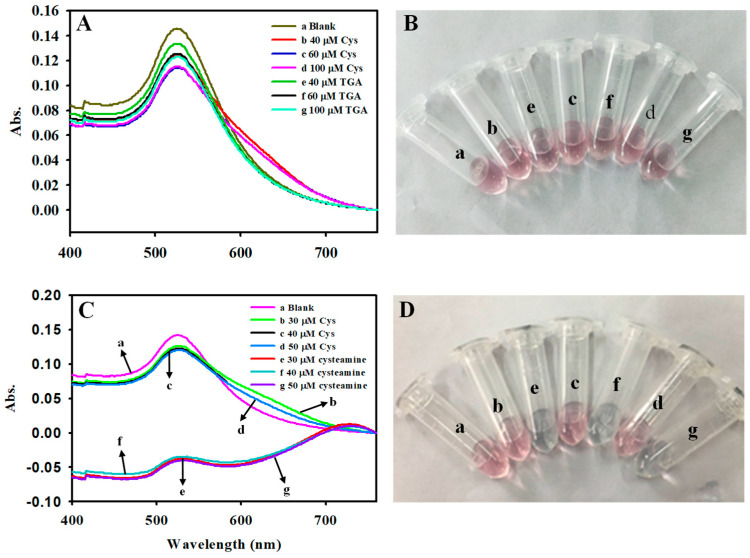
UV–vis spectra (**A**) and corresponding photographs (**B**) of AuNPs–Cys and AuNPs–TGA mixed solution with various concentrations of Cys and TGA. UV–vis spectra (**C**) and corresponding photographs (**D**) of AuNPs–Cys and AuNPs–cyseamine mixed solution with various concentrations of Cys and cyseamine.

**Figure 2:**
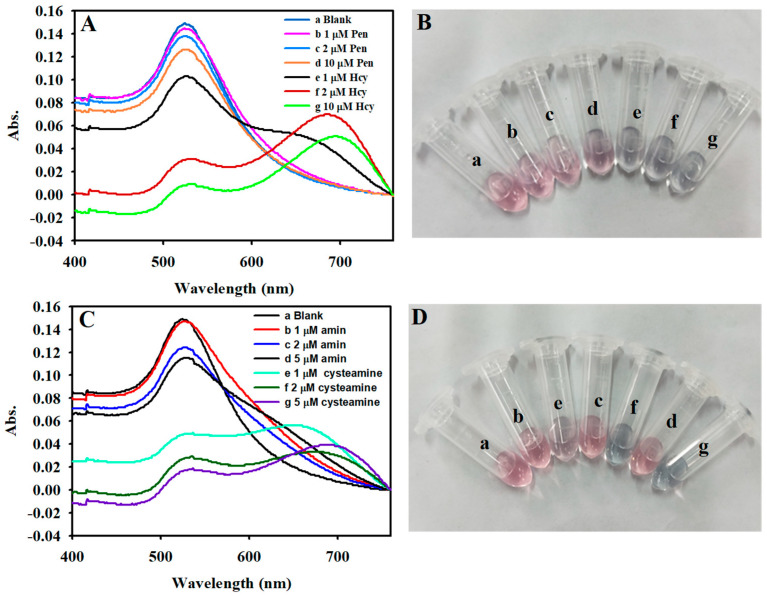
UV–vis spectra (**A**) and corresponding photographs (**B**) of AuNPs–Pen and AuNPs–Hcy mixed solution with various concentrations of Hcy and Pen. UV–vis spectra (**C**) and corresponding photographs (**D**) of AuNPs–amin and AuNPs–cysteamine mixed solution with various concentrations of amin and cysteamine.

**Figure 3:**
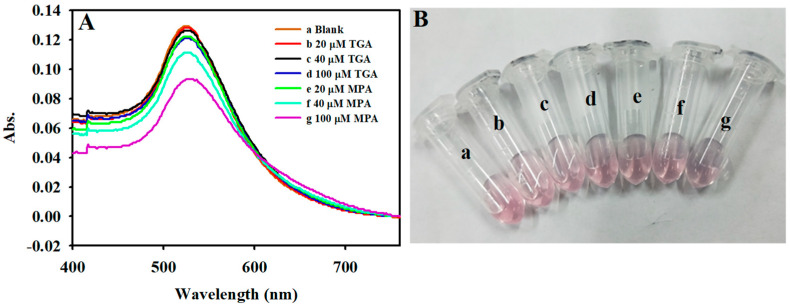
UV–vis spectra (**A**) and corresponding photographs (**B**) of AuNPs–TGA and AuNPs–MPA mixed solution with various concentrations of TGA and MPA.

First, we designed two control groups to investigate the role of amino and carboxyl groups in AuNPs’ aggregation induced by Hcy and Cys molecule. Herein, we selected the same concentration of TGA and Cys (excluding amino, as a control) for a comparison of AuNPs’ aggregation. As shown in Figure 1A, the aggregation effect of Cys on AuNPs is stronger than mercaptoacetic acid, with a more apparent color change (Figure 1B). It is suggested that when amino and carboxyl groups are present at the same time, it may be that the amino group plays a major role in the aggregation of AuNPs, and it is also possible that the amino group and the carboxyl group together contribute to the aggregation of AuNPs. In order to further confirm the role of amino and carboxyl groups in the aggregation of AuNPs, we chose the same concentration of cyseamine and Cys (excluding carboxyl, as a control) as a comparison of AuNPs’ aggregation. As shown in Figure 1C, cyseamine has a stronger aggregation effect on AuNPs than Cys, with a more apparent color change (Figure 1D), which indicated that the amino group is responsible for the aggregation of AuNPs. In addition, we suspected that the different steric hindrance caused by the different molecular structures would affect the AuNPs’ aggregation. To verify this conjecture, we compared the same concentration of Pen (with large steric hindrance) and Hcy for the aggregation of AuNPs. As shown in Figure 2A,B, the effect of the concentration of Hcy on AuNPs was obviously stronger than that of Pen, suggesting that a molecule with less steric hindrance was more beneficial to the aggregation of AuNPs. To further discuss the important role of steric hindrance in the aggregation of AuNPs, we chose the same concentration of amin (with large steric hindrance) and cyseamine for AuNPs’ aggregation as a comparison. As shown in Figure 2C,D, the effect of cyseamine on AuNPs was significantly stronger than that of amin, further indicating that molecules with small space resistance are more favorable for AuNPs’ aggregation. It was found that even over 40 min, the color change of AuNPs can hardly be observed after the addition of Pen (low concentration), amin, and Cys, while all of these changes could be observed within 10 min after the addition of cyseamine or Hcy. These results indicated that the steric hindrance effect plays an important role here. Finally, the effect of carbon chain length on the aggregation of AuNPs is verified. Here, the same concentrations of TGA and MPA were used as control experiments to investigate the effect of carbon chain length on AuNPs’ aggregation. As shown in Figure 3, the aggregation effect of MPA on AuNPs is stronger than that of TGA, demonstrating that the carbon chain length also affects the aggregation effect of AuNPs. Similarly, the different length of the carbon chain between Cys and Hcy (only one, –CH_2_–, is different) is also a factor for the different aggregation effects of Cys and Hcy on AuNPs. Moreover, the amino group plays a major role in AuNPs’ aggregation in the presence of both amino and carboxyl groups, while, on the contrary, the effect of the carboxyl group on AuNPs’ aggregation was a steric hindrance effect, which dampens AuNPs’ aggregation effect. All of the above results show that highly selective detection of Hcy can be explained based on the results of synergy between hydrogen bonding, steric hindrance, and carbon chain length in Hcy and Cys with AuNPs.

### 3.4. Optimization for Hcy Detection

To obtain high detection sensitivity, several relevant experimental parameters, such as the pH values of the solution and the incubation time, were investigated. Firstly, pH is one of the most necessary factors in our work above all, because it may influence the interaction between Hcy and AuNPs, as well as the self-aggregation of AuNPs. Thus, we carried out Hcy detection in four frequently used buffers (citrate buffer, PBS buffer, citric acid–disodium phosphate buffer, and BR buffer) at 5 mM concentrations. Considering the effect of pH on self-aggregation of AuNPs and interaction between Hcy and AuNPs, the optimal buffer was obtained in PBS buffer (pH 5.0) (Figures S6–S9). Therefore, a 5 mM PBS buffer solution with pH 5.0 was used throughout this study (Figure S10A). In our experiments, the absorbance difference was used as a criterion for selecting the best conditions. The absorbance difference is defined as A_0_ − A_1_, and A_0_ and A_1_ represent the absorbance in the absence and presence of Hcy. In addition, we also investigated the effect of incubation time on the absorbance difference (A_0_ − A_1_). As presented in Figure S10B, when the incubation time reaches 20 min, the absorbance difference remains stable. Therefore, the ideal incubation time for Hcy detection is 20 min.

### 3.5. Sensitive Detection of Hcy

**Figure 4:**
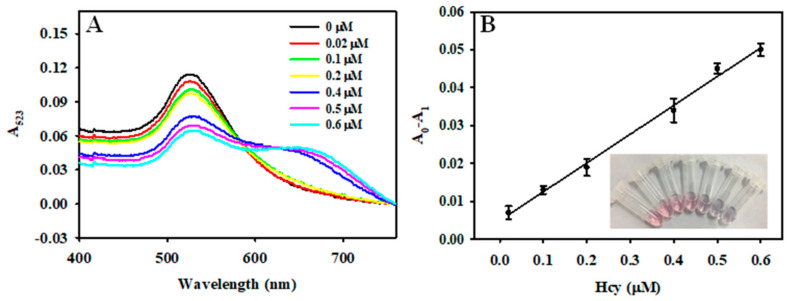
(**A**) The absorbance spectra of the AuNP solution in the presence of different concentrations of Hcy (from 0 to 0.6: 0, 0.02, 0.1, 0.2, 0.4, 0.5, and 0.6 μM). (**B**) The related linear relationship towards different concentrations of Hcy. [AuNPs]: 1 nM.

Under optimal conditions, the capability of the analytical system to quantify Hcy was assessed. As shown in Figure 4A, the absorbance of AuNPs at 523 nm is gradually diminished (from top to bottom) with increasing Hcy concentration, which indicates that the change in absorbance and Hcy concentration is dose-related. The absorbance difference is found to be linear with the concentration of Hcy in the range of 0.02 to 0.6 μM (Figure 4B), and the calibration curve can be expressed as A_0_ − A_1_ = 0.005 + 0.076 C (C is the concentration of Hcy), with a correlation coefficient (R^2^) of 0.995. The limit of detection (LOD) is 0.008 μM (S/N = 3), which is lower than the detection limit of most reported methods (Table 1). The low detection concentration and the high response effect for Hcy allowed us to smartly identify the existence of trace quantities of Hcy in serum samples.

### 3.6. Specificity for the Detection of Hcy

As we all know, the main barrier to Hcy detection comes from some amino acids and similar chemical structures of thiol molecules, such as Cys and others. Prevention of the interference of structurally similar molecules is reasonably expected in the design of a highly selective Hcy-related sensor. To evaluate the selectivity of our development methods, some potential interfering substances, including common cations, 16 kinds of amino acids, and biological thiols, were tested for their effects on Hcy detection under the same conditions. As shown in Figure S11, the AuNP system exhibits a negligible absorbance change in relation to other metal ions and sixteen amino acids. In particular, the addition of 40 times the amount of Cys and GSH did not affect AuNP absorbance changes. Therefore, these potential interfering substances do not affect the detection of Hcy. The discrimination effect for Hcy originates from the interaction difference of the hydrogen bonding, steric hindrance, and carbon chain length in Hcy and Cys with AuNPs. Reports of highly selective detection of Hcy are extremely rare, as far as we know. All of these results clearly show that the designed method is highly selective for distinguishing between Hcy and other related substances.

### 3.7. Hcy Detection in Human Serum Samples

We further applied this highly sensitive and selective approach to the practical analysis of the amount of Hcy in human serum through the standard addition method. Taking into account the Hcy level in healthy human serum and the linear range of our method, the serum samples were diluted four-fold before testing. As shown in Table S1, the recoveries of Hcy reached up to 93.8–109.1%, with a relative standard deviation (RSD) of 0.01–0.37%. These results imply that our method can sensitively detect Hcy in complex serum samples. In order to further validate the accuracy of the proposed method, one clinical method based on an enzymatic assay was used to measure Hcy in the same serum sample. The analytical results obtained through the developed method agreed closely with the clinical data provided by the local hospital (Table 2), unambiguously indicating that our proposed method is an effective and reliable technology for the analysis of Hcy in serum samples.

## 4. Conclusions

In conclusion, we have developed a novel and simple method to detect Hcy with high selectivity and sensitivity using AuNPs. Owing to the unique and strong thiol–Au interaction, the AuNPs showed superior selectivity for Hcy over the other 16 natural amino acids. In particular, the method is able to implement the selective detection of Hcy in the presence of a large number of Cys (Cys higher than 40 times Hcy), in accordance with the slight differences between the two structures (only one, –CH_2_–, is different). In addition, the sensing system can also be used to detect Hcy from real serum samples, and the results were consistent with enzymatic-based clinical methods. Furthermore, the assay is of interest not only because it provides a simple and fast Hcy sensor with high selectivity and sensitivity but also because it exemplifies the utilization of the physical property (hydrogen bonding and steric hindrance) of the target analyte on the nanoparticles’ or nanoclusters’ (NCs) surface, which is, however, missing in the current construction of sensors by using nanoparticles or NCs.

## Figures and Tables

**Table 1:** Comparison of different methods for the determination of Hcy.

Methods	Linear Range (μM)	Detection Limit (μM)	Reference
Electrochemistry	0.02–20	0.017	[27]
Electrochemistry	1–50	0.22	[28]
Fluorometry	0–50	0.06	[12]
Fluorometry	0–500	2.7	[13]
Fluorometry	0–25	0.11	[15]
Fluorometry	0–90	1.9	[16]
Colorimetry	3.0–20	0.4	[29]
Colorimetry	2.5–50	1.48	[30]
Fluorometry	0.02–0.6	0.008	Present work

**Table 2:** Detection of Hcy in serum samples.

Samples	Medical Examination (μM)	Measured (μM)	RSD (n = 3, %)
1	14.1	13.5	0.17%
2	16.1	13.5	0.50%
3	10.1	9.7	0.30%
4	9.3	8.5	0.17%
5	8.9	8.4	0.11%
6	11	11	0.60%
7	8.7	7.2	0.20%
8	8.2	7.6	0.21%
9	15.1	14.8	0.17%
10	11.5	12.2	0.49%

## Data Availability

Data are contained within the article and Supplementary Materials.

## Supplementary Materials

The following supporting information can be downloaded at https://www.mdpi.com/article/10.3390/molecules30061309/s1, Figure S1: TEM images of AuNPs with different sizes; Figure S2. (A) UV-vis absorption spectra of AuNPs (a), AuNPs/Cys (40 μM) (b), AuNPs/Hcy (1 μM) (c). (B) UV-vis absorption spectra of AuNPs (a), AuNPs/Cys (40 μM) (b), AuNPs/Hcy (1 μM) (c); Figure S3. TEM images (A), size distribution histograms (B) of AuNPs; Figure S4. The absorption spectrum of (a) AuNPs, (b) AuNPs + Cys (40 µM), (c) AuNPs + Hcy (1 µM), respectively. [AuNPs]: 1 nM; Figure S5. The Zeta potential of AuNPs; Figure S6. Effect of different pH values on the detection of Hcy with AuNPs; Figure S7. Effect of different pH values on the detection of Hcy with AuNPs; Figure S8. Effect of different pH values on the detection of Hcy with AuNPs; Figure S9. Effect of different pH values on the detection of Hcy with AuNPs; Figure S10. (A) Bar graph of UV-vis responses to the different buffers (BR (5 mM, pH 5.0), PBS (5 mM, pH 5.0), C-P buffer (crtric acid-disodium phosphate 5 mM, pH 5.0), Critrate (5 mM, pH 5.0)). (B) Effects of incubation time on the UV-vis responses sensor for Hcy detection; Figure S11. The interfering effects of relevant substances on the AuNPs nanosensor for detection of Hcy. The relevant substances are: (a) blank; (b) glycine; (c) NaCl; (d) ZnCl_2_; (e) KCl; (f) mannose; (g) serine; (h) asparagine; (i) argnine; (j) glutamic acid; (k) lysine; (l) tryptophane; (m) dopamine hydrochloride; (n) cystine; (o) threonine; (p) hlstidine; (q) alanine; (r) proline; (s) glucose; (t) GSH; (u) Cys; (v) Hcy. The concentration of Hcy was 0.5 μM, and the concentrations of other possible interferences are 20 μM; Table S1. The application of the method for determination of serum sample with different amounts of Hcy.
