# Supplementary material for: Smart Probes for Ultrasensitive and Highly Selective Sensing of Homocysteine over Cysteine Based on Multi-Cooperative Effects by Using Gold Nanoparticles
Source: Molecules. 2025 Mar 14;30(6):1309. doi: 10.3390/molecules30061309 (PMC11946112; doi:10.3390/molecules30061309)
Supplement: Supplementary file 1 [file molecules-30-01309-s001.zip › molecules-3473536-supplementary.pdf]

Supporting Information

Smart Probes for Ultrasensitive and Highly Selective Sensing of Homocysteine over Cysteine Based on Multi-cooperative Effects by Using Gold Nanoparticles

Manman Sun [1], Peihao Zhang [1], Zeze Xie [2], Pengcheng Zhang [2], Zhendong Li [2], Zhiguang Yang [2] and Hongyu Chen [2,*]

[1] College of Physics and Telecommunication Engineering, Zhoukou Normal University, Zhoukou 466001, China

[2] Henan Key Laboratory of Rare Earth Functional Materials, College of Chemistry and Chemical Engineering, Zhoukou Normal University, Zhoukou 466001, China

*Corresponding author. Tel: +86-394-8178518; fax: +86-394-8178518;

Email address: chy199166@163.com

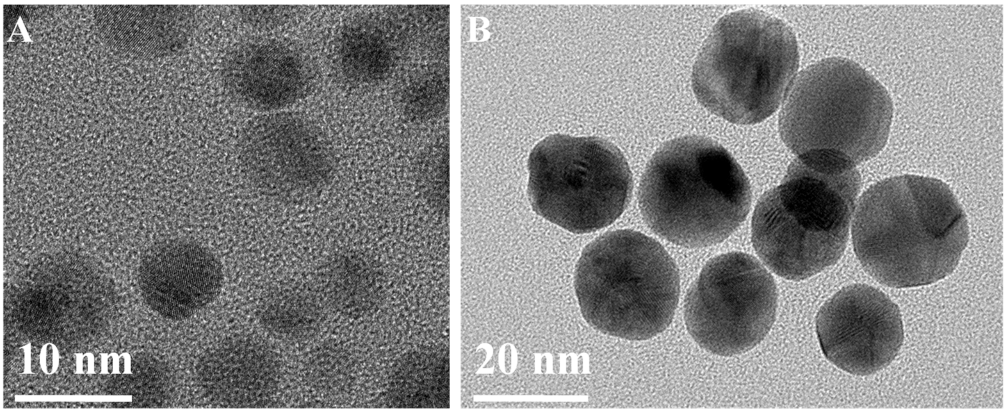

Figure S1. TEM images of AuNPs with different sizes.

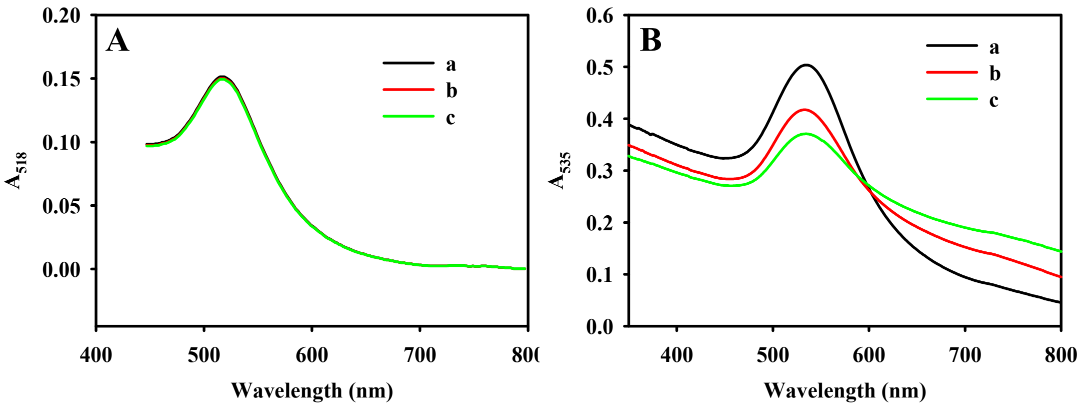

Figure S2. (A) UV-vis absorption spectra of AuNPs (a), AuNPs/Cys (40 μM) (b), AuNPs/Hcy (1 μM) (c). (B) UV-vis absorption spectra of AuNPs (a), AuNPs/Cys (40 μM) (b), AuNPs/Hcy (1 μM) (c).

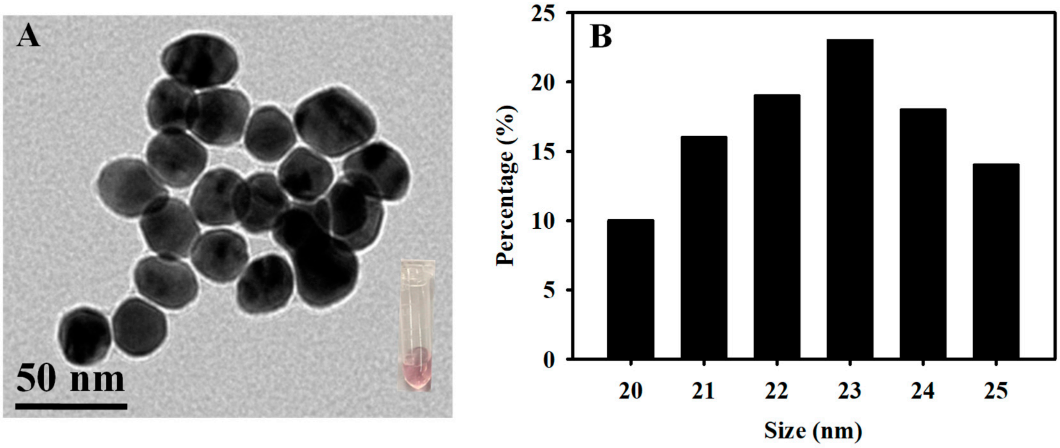

Figure S3. TEM images (A), size distribution histograms (B) of AuNPs.

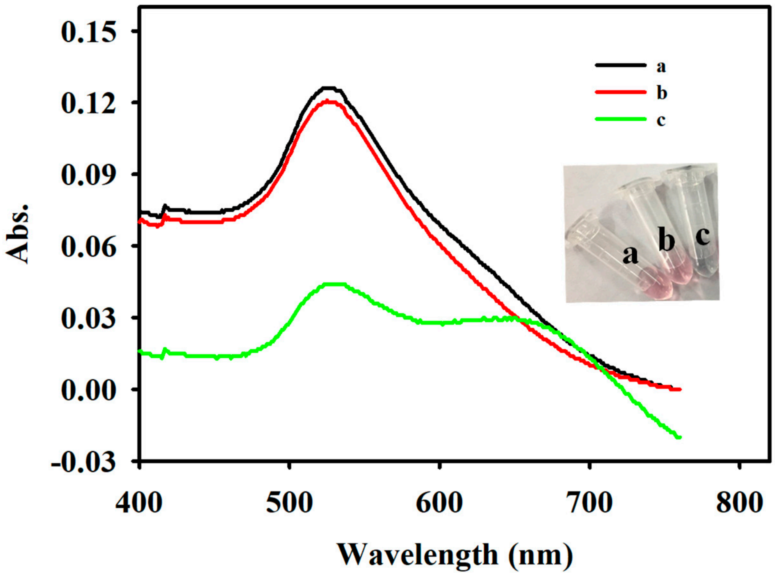

Figure S4. The absorption spectrum of (a) AuNPs, (b) AuNPs + Cys (40 μM), (c) AuNPs + Hcy (1 μM), respectively. [AuNPs]: 1 nM.

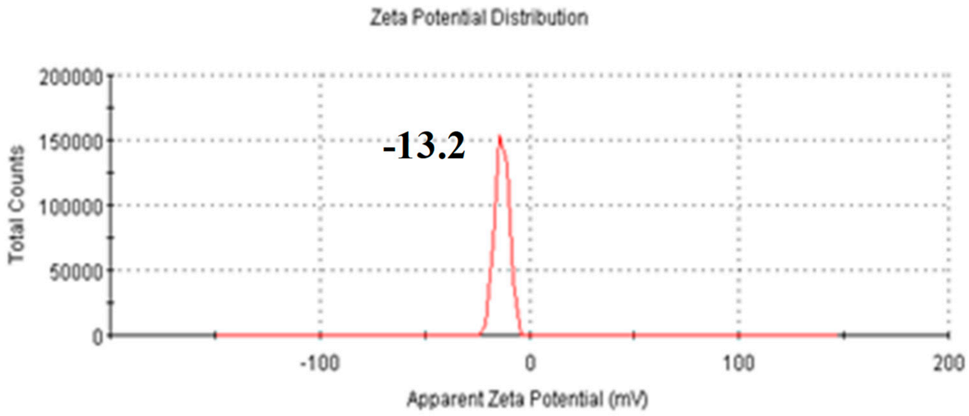

Figure S5. The Zeta potential of AuNPs.

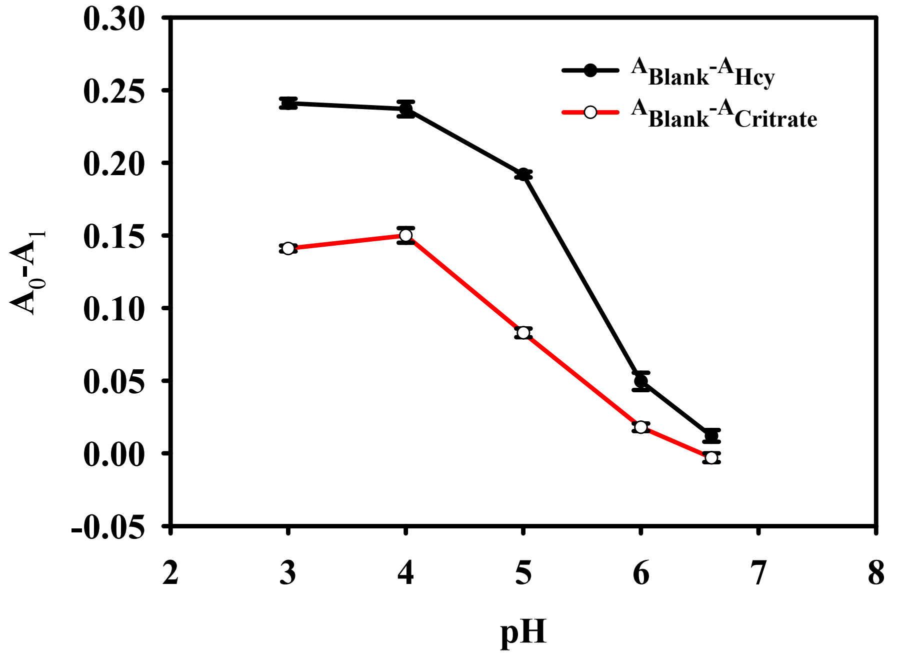

Figure S6. Effect of different pH values on the detection of Hcy with AuNPs.

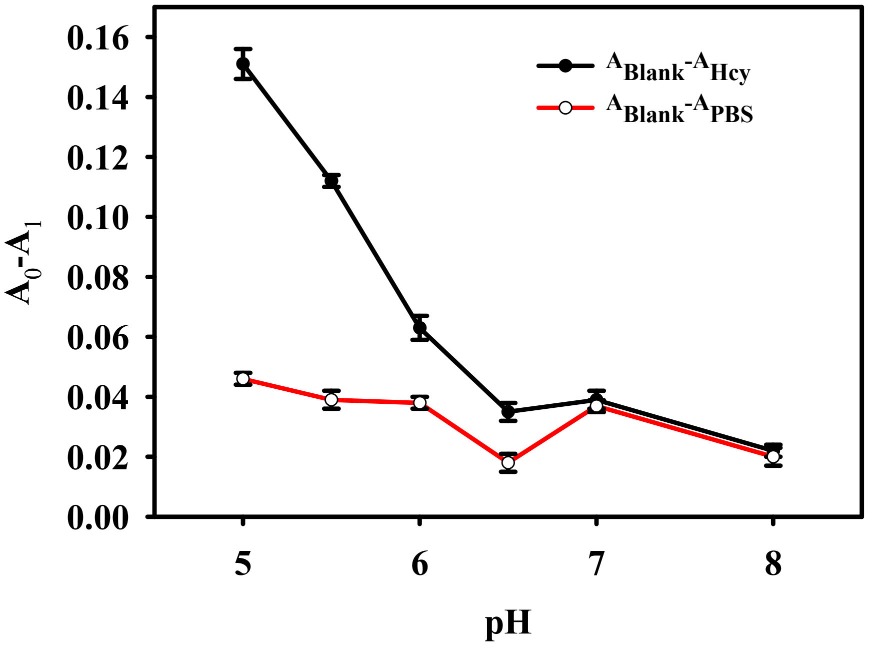

Figure S7. Effect of different pH values on the detection of Hcy with AuNPs.

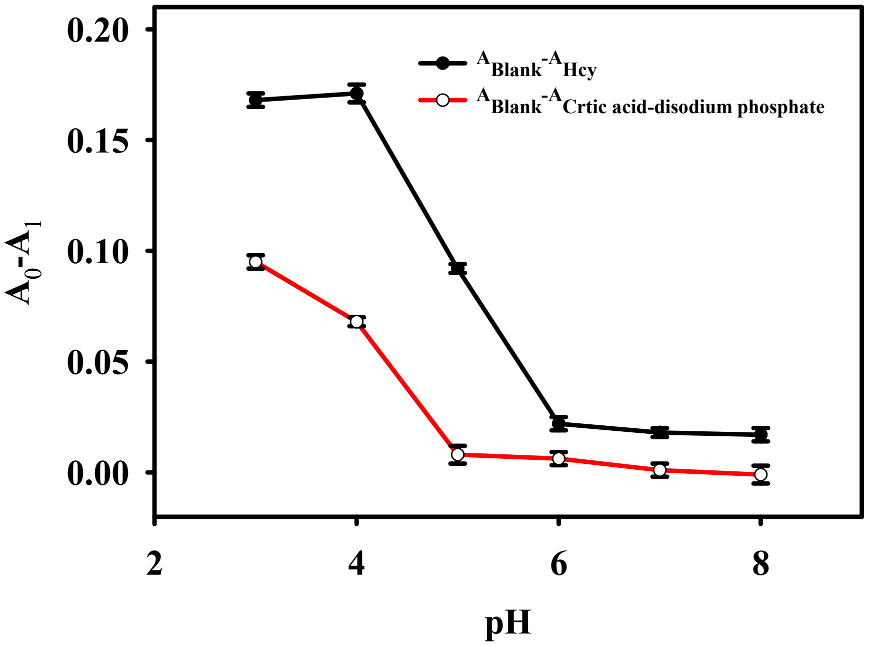

Figure S8. Effect of different pH values on the detection of Hcy with AuNPs.

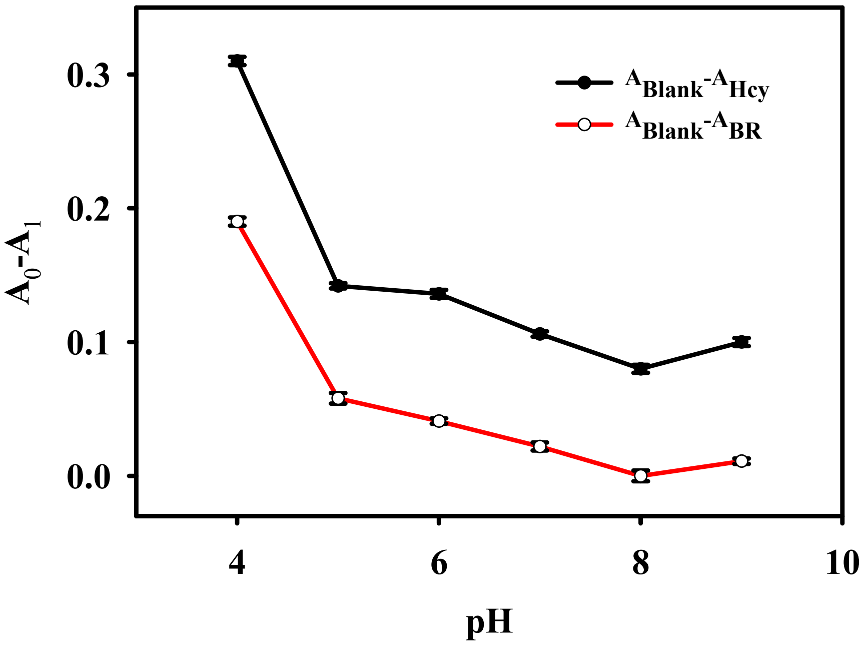

Figure S9. Effect of different pH values on the detection of Hcy with AuNPs.

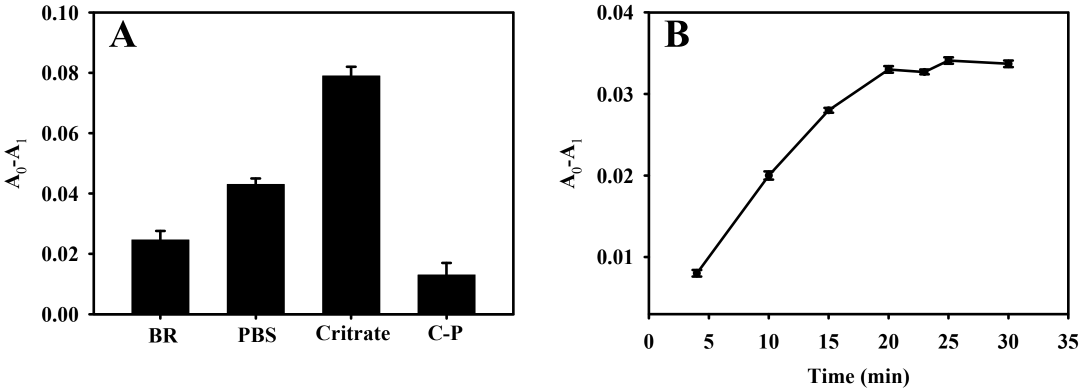

Figure S10. (A) Bar graph of UV-vis responses to the different buffers (BR (5 mM, pH 5.0), PBS (5 mM, pH 5.0), C-P buffer (crtric acid-disodium phosphate 5 mM, pH 5.0), Critrate (5 mM, pH 5.0)). (B) Effects of incubation time on the UV-vis responses sensor for Hcy detection.

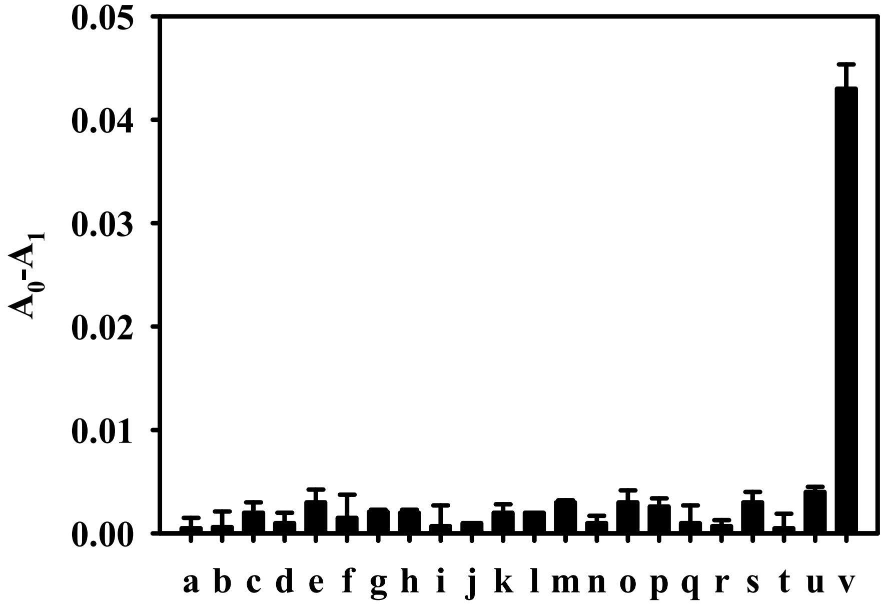

Figure S11. The interfering effects of relevant substances on the AuNPs nanosensor for detection of Hcy. The relevant substances are: (a) blank; (b) glycine; (c) NaCl; (d) $ZnCl_2$; (e) KCl; (f) mannose; (g) serine; (h) asparagine; (i) argnine; (j) glutamic acid; (k) lysine; (l) tryptophane; (m) dopamine hydrochloride; (n) cystine; (o) threonine; (p) hlstidine; (q) alanine; (r) proline; (s) glucose; (t) GSH; (u) Cys; (v) Hcy. The concentration of Hcy was 0.5 μM, and the concentrations of other possible interferences are 20 μM.

Table S1. The application of the method for determination of serum sample with different amounts of Hcy.

Sample	Added (μM)	Found (μM)	Recovery (%)	RSD (n=3, %)
Serum 1	-	0.06	-	0.01
	0.05	0.119	109	0.01
	0.1	0.15	94	0.01
	0.4	0.216	102	0.37
